# Comparative Genomic Analysis Reveals Novel Microcompartment-Associated Metabolic Pathways in the Human Gut Microbiome

**DOI:** 10.3389/fgene.2019.00636

**Published:** 2019-07-04

**Authors:** Dmitry A. Ravcheev, Lubin Moussu, Semra Smajic, Ines Thiele

**Affiliations:** ^1^School of Medicine, National University of Ireland, Galway, University Road, Galway, Ireland; ^2^Luxembourg Centre for Systems Biomedicine, University of Luxembourg, Esch-sur-Alzette, Luxembourg; ^3^Discipline of Microbiology, School of Natural Sciences, National University of Ireland, Galway, University Road, Galway, Ireland

**Keywords:** human gut microbiome, comparative genomics, bacterial microcompartments, metabolic reconstruction, metabolosome

## Abstract

Bacterial microcompartments are self-assembling subcellular structures surrounded by a semipermeable protein shell and found only in bacteria, but not archaea or eukaryotes. The general functions of the bacterial microcompartments are to concentrate enzymes, metabolites, and cofactors for multistep pathways; maintain the cofactor ratio; protect the cell from toxic metabolic intermediates; and protect the encapsulated pathway from unwanted side reactions. The bacterial microcompartments were suggested to play a significant role in organisms of the human gut microbiome, especially for various pathogens. Here, we used a comparative genomics approach to analyze the bacterial microcompartments in 646 individual genomes of organisms commonly found in the human gut microbiome. The bacterial microcompartments were found in 150 (23.2%) analyzed genomes. These microcompartments include previously known ones for the utilization of ethanolamine, 1,2-propanediol, choline, and fucose/rhamnose. Moreover, we reconstructed two novel pathways associated with the bacterial microcompartments. These pathways are catabolic pathways for the utilization of 1-amino-2-propanol/1-amino-2-propanone and xanthine. Remarkably, the xanthine utilization pathway does not demonstrate similarity to previously known microcompartment-associated pathways. Thus, we describe a novel type of bacterial microcompartment.

## Introduction

Bacterial microcompartments (BMCs) are self-assembling subcellular structures and analogs of eukaryotic organelles. Unlike eukaryotic organelles, BMCs are surrounded not by a lipid membrane but by a semipermeable protein shell (Kerfeld et al., 2010; Kerfeld et al., 2018). The BMCs are icosahedral structures with a diameter of 100–150 nm consisting of 10–20 types of polypeptides (Lawrence et al., 2014). The total number of polypeptides in a single BMS is estimated to be approximately 10,000 to 20,000 (Murat et al., 2010). These polypeptides include shell proteins and encapsulated enzymes. The general functions of BMCs are as follows: 1) the concentration of enzymes, metabolites, and cofactors for multistep pathways; 2) maintenance of the cofactor ratio; 3) protection of the cell from toxic metabolic intermediates; and 4) protection of the encapsulated pathway from unwanted side reactions (Cheng et al., 2008; Kerfeld et al., 2010; Murat et al., 2010;Kerfeld et al., 2018).

The protein shell of BMCs comprises three types of proteins: BMC-H, BMC-P, and BMC-T. BMC-H is the most abundant type, containing a single Pfam00936 domain and forming a cyclic hexamer (Yeates et al., 2013; Sutter et al., 2017). BMC-T proteins contain two Pfam00936 (Plegaria and Kerfeld, 2017) domains as a tandem repeat and form cyclic pseudohexamers, i.e., trimers of two-domain proteins (Plegaria and Kerfeld, 2017). BMC-P proteins contain a single Pfam03319 and form pentamers. BMC-H and BMC-P proteins constitute the facets of the BMC shells, whereas BMC-P proteins constitute the vertices of the icosahedral shell (Plegaria and Kerfeld, 2017). A pore, formed at the central symmetry axis of the BMC-H hexamers and BMC-T pseudohexamers, serves as a channel for metabolites. Thus, the BMC-H and BMC-T proteins determine the permeability of the BMC shell for specific metabolites (Mallette and Kimber, 2017; Sutter et al., 2017)

**Figure 1:**
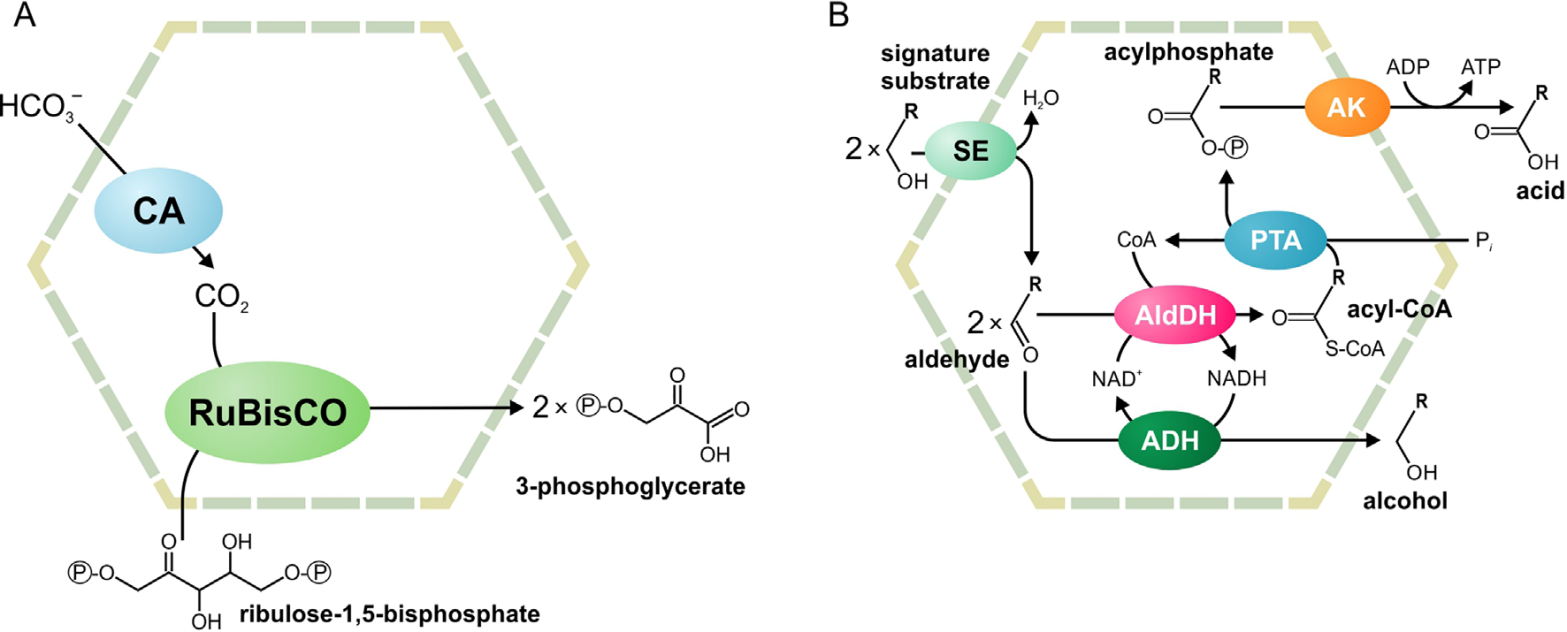
Schematic illustration of the carboxysome **(A)** and metabolosome **(B)** organization. ADH, alcohol dehydrogenase; AldDH, aldehyde dehydrogenase; AK, acetyl kinase; CA, carbonic anhydrase; PTA, phospotransacylase; RuBisCO, ribulose-1,5-bisphosphate carboxylase/oxygenase; SE, signature enzyme.

Two main functional paradigms have emerged: anabolic carboxysomes and catabolic metabolosomes. Carboxysomes are CO_2_-fixing BMCs found in cyanobacteria and some proteobacteria (**Figure 1A**). These BMCs encapsulate the enzymes carbonic anhydrase and D-ribulose 1,5-bisphosphate carboxylase/oxygenase (RuBisCO). Carbonic anhydrase converts HCO_3_^-^ to CO_2_. Subsequently, RuBisCO converts CO_2 _and ribulose bisphosphate to two molecules of 3-phosphoglycerate. The shell of the carboxysome is permeable to charged molecules, such as HCO_3_^-^, ribulose bisphosphate, and 3-phosphoglycerate, but restricts diffusion of CO_2_. An additional function of the carboxysome is the prevention of unwanted side reactions, such as the oxygenation of D-ribulose 1,5-bisphosphate. Such a reaction can occur in the cytoplasm when RuBisCO uses O_2_ instead of CO_2_. As the carboxysome shell restricts diffusion of O_2_, this reaction is prevented (Cheng et al., 2008; Tanaka et al., 2009; Rae et al., 2013; Aussignargues et al., 2015; Turmo et al., 2017).

**Figure 2:**
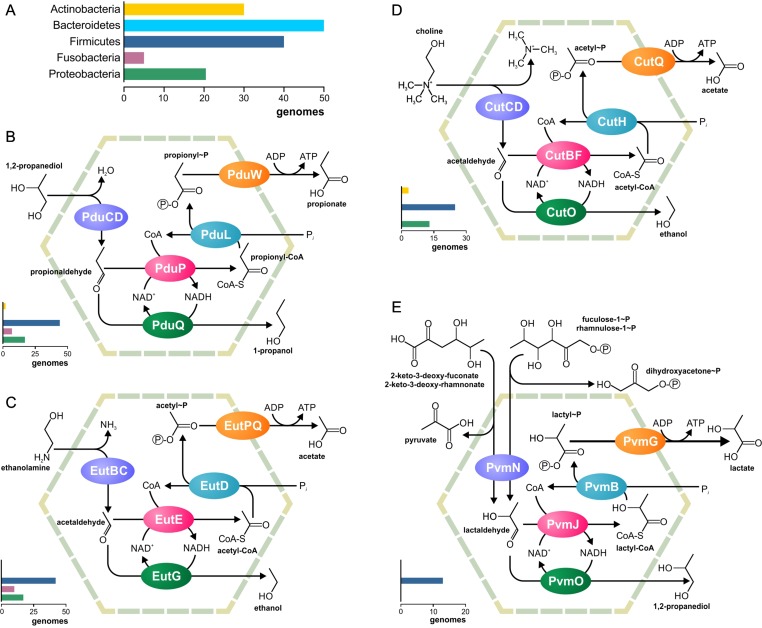
Metabolic pathways associated with the previously known BMCs. **(A)** Legend for the chart on the phyletic distribution of the pathways and numbers of genomes with the BMC are shown. The chart demonstrates the number of analyzed genomes, in which this pathway is present. **(B–E)** Analyzed metabolic pathways and their phyletic distribution. BMC pathways for utilization of 1,2-propanediol **(B)**, ethanolamine **(C)**, choline **(D)**, and fucose/rhamnose **(E)** are shown.

Metabolosomes are catabolic compartments that encapsulate enzymes of different pathways and share a common core biochemistry (**Figure 1B**). In a first step, the input, or *signature substrate,* is converted into an aldehyde. For the correct function of the metabolosome, two molecules of a signature substrate are transformed into two molecules of the corresponding aldehyde. One molecule of the aldehyde is then converted to an alcohol by the alcohol dehydrogenase, while oxidizing one molecule of NADH to NAD^+^. In a parallel reaction, another molecule of the aldehyde is converted to acyl-CoA, reducing one molecule of NAD^+^ to NADH. Acyl-CoA is then phosphorylated to an acylphosphate by the phospotransacylase with the release of HS-CoA. The acylphosphate permeates the BMC shell and is dephosphorylated by a kinase in an ATP-generating reaction (**Figure 1B**). The last reaction takes place outside the BMC but genes for the kinase are often located in the BMC gene clusters (Axen et al., 2014; Lawrence et al., 2014; Kerfeld and Erbilgin, 2015; Erbilgin et al., 2016). Thus, the metabolosome combines the following functions: 1) the concentration of enzymes and metabolites for the catabolic pathway, 2) protection of the cell from aldehyde intermediates, and 3) recycling of cofactors, acyl-CoA/HS-CoA, and NAD^+^/NADH, thus making a pathway independent of the concentrations of these cofactors in the cytoplasm. Until now, metabolosomes have been identified for the utilization of four different signature substrates (**Figure 2**): 1,2-propanediol (Bobik et al., 1997; O’brien et al., 2004; Sriramulu et al., 2008;Parsons et al., 2010; Fan and Bobik, 2011; Zarzycki et al., 2017; Levin and Balskus, 2018), ethanolamine (Tsoy et al., 2009; Srikumar and Fuchs, 2011; Kendall et al., 2012; Pitts et al., 2012), choline (Martinez-Del Campo et al., 2015; Craciun et al., 2016; Backman et al., 2017), and fucose/rhamnose (Petit et al., 2013; Erbilgin et al., 2014).

One additional BMC has been exclusively found in the strict anaerobe *Clostridium kluyveri*. The gene cluster for this BMC encodes two different shell proteins, two copies of an aldehyde dehydrogenase, and three copies of an alcohol dehydrogenase. This BMC has been suggested to be involved in acetate/ethanol oxidation into acetyl-CoA, but the proposed pathway does not allow the recycling of cofactors. Thus, this BMC still requires better biochemical and genetic characterization (Seedorf et al., 2008; Heldt et al., 2009).

The BMCs have been suggested to significantly influence organisms of the human gut microbiome (HGM), especially various pathogens (Jakobson and Tullman-Ercek, 2016). The BMC genes have been found in the genomes of multiple human pathogens, such as pathogenic strains of *Escherichia coli*, *Listeria monocytogenes*, *Salmonella enterica*, *Shigella flexneri*, and *Yersinia enterocolitica* (Fan et al., 2012; Axen et al., 2014; Kerfeld et al., 2018). Two signature substrates for metabolosomes, 1,2-propanediol and ethanolamine, have been associated with food poisoning (Korbel et al., 2005). Additionally, ethanolamine utilization by the metabolosome allows *S. enterica* to compete with healthy gut microbiota (Thiennimitr et al., 2011). Trimethylamine, a product of choline degradation by the metabolosome, has been associated with cardiovascular (Rath et al., 2017; Ashikhmin et al., 2018; Tripathi et al., 2018) and kidney (Moraes et al., 2015; Tang et al., 2015) diseases.

Comparative genomics studies have been already successfully used to analyze the BMCs in various microbial genomes (Tsoy et al., 2009; Jorda et al., 2013; Axen et al., 2014; Zarzycki et al., 2015). In this study, we targeted exclusively HGM genomes and analyzed them by combining phylogenomic and genome context-based techniques (Osterman and Overbeek, 2003; Rodionov, 2007). This approach has been repeatedly used to reconstruct other metabolic pathways, including respiration (Ravcheev and Thiele, 2014), the biosynthesis of B-vitamins (Magnusdottir et al., 2015), and quinones (Ravcheev and Thiele, 2016), as well as central carbon metabolism and the biosynthesis of amino acids and nucleotides (Magnusdottir et al., 2017). By focusing on HGM genomes, we were able to characterize the distribution of known BMCs in the HGM as well as also to reconstruct two novel BMC-associated pathways to utilize 1-amino-2-propanol/1-amino-2-propanone and xanthine. Remarkably, the xanthine utilization pathway does not demonstrate similarity with pathways for carboxysomes or metabolosomes, thus creating a third functional paradigm for the BMCs.

## Methods

### Analyzed Genomes

The set of analyzed HGM genomes includes the following: 1) 633 genomes from the AGORA resource (Magnusdottir et al., 2017), which are available at the PubSEED database (Overbeek et al., 2005; Disz et al., 2010), 2) eight genomes with genes for the novel 1-amino-2-propanol/1-amino-2-propanone utilization BMC (see the section Amino-2-Propanol/1-Amino-2-Propanone Utilization), and five genomes with genes for the novel xanthine utilization BMC (see the section Xanthine Utilization). Of these 646 genomes, 609 had a finished sequencing status, whereas 37 had a draft status (**Supplementary Table S1**). These genomes represent 559 microbial species, 165 genera, 76 families, 31 orders, 20 classes, and 12 phyla. All the selected genomes are bacterial with the exception of three archaea. The phyletic distribution of the analyzed genomes is in good agreement with previously reported HGMs (Eckburg et al., 2005; Goodman et al., 2011; Walker et al., 2011; Graf et al., 2015), i.e., the most represented phyla are Actinobacteria (99 genomes, 15.3% of the analyzed genomes), Bacteroidetes (97 genomes, 15%), Firmicutes (299 genomes, 46.3%), and Proteobacteria (123 genomes, 19%).

### Comparative Genomics Approach

We used a comparative genomics approach to annotate BMC genes in the analyzed genomes. Two main directions were used for the annotation: 1) the search of orthologs of the known BMC proteins, i.e., enzymes and shell proteins (**Supplementary Table S2**) and 2) the search of homologs of known shell proteins. Orthologs were defined as the best bidirectional hits (BBHs) with a similar genomic context. The BBHs are required to have: a score ≥150 bits, an e-value ≤e^-50^, a protein identity and positives ≥30% and ≥50%, respectively, and a query coverage of ≥70%. The similar genomic context was determined as follows: 1) The BBH genes formed an operon or divergon, i.e., a pair of divergently transcribed operons in more than one microbial species. 2) Genes were considered to form an operon if they had the same direction and distance between two adjacent genes, which did not exceed 100 bp. 3) A pair of operons was considered a divergon if they were divergently transcribed and the distance between the starts of the operons did not exceed 400 bp. 4) The start of the operon was determined as the start of translation of the first gene of a predicted operon.

For homologs of the BMC shell, the following parameters were used: a score ≥20 bits, an e-value ≤e^-5^, a protein identity and positives ≥30% and ≥50%, respectively, and a query coverage of ≥40%. These parameters were selected after comparing the known BMC proteins. Because these weakened parameters for a search of homologs of the shell proteins could result in a large number of false-positive results, all the identified orthologs were checked for the presence of signature domains for the shell BMC proteins (Zarzycki et al., 2015; Plegaria and Kerfeld, 2017). Thus, only proteins having domains characteristic of BMCH/BMCT (Pfam00936) or BMCP (Pfam03319) proteins were considered BMC shell proteins.

For analysis of the proteins evolution for 1-amino-2-propanol/1-amino-2-propanone utilization (see the section Evolution of the 1-Amino-2-Propanol/1-Amino-2-Propanone Utilization), homologs were searched in all genomes using the parameters: a score ≥100 bits, an e-value ≤e^-30^, and a protein identity ≥30%.

### Tools and Databases

The PubSEED platform (Overbeek et al., 2005; Disz et al., 2010) was used to annotate the BMC proteins. To search for BBHs for previously known proteins, a BLAST algorithm (Altschul et al., 1997) implemented in the PubSEED platform was used. The same algorithm was also used to search for homologs of the BMC shell proteins. Additionally, the PubSEED platform was used to predict the operons and divergons. To analyze the protein domain structure, we searched the Conserved Domains Database (CDD) (Marchler-Bauer et al., 2013) using the following parameters: an e-value ≤0.01 and a maximum number of hits equal to 500.

Alignments were performed using MUSCLE v.3.8.31 (Edgar, 2004a; Edgar, 2004b). For every multiple alignment, position quality scores were evaluated using Clustal X (Thompson et al., 1997; Larkin et al., 2007). Thereafter, all positions with a score of zero were removed from the alignment and the modified alignment was used for construction of the phylogenetic trees. Phylogenetic trees were constructed using the maximum-likelihood method with the default parameters implemented in PhyML-3.0 (Guindon et al., 2010). The obtained trees were midpoint-rooted and visualized using the interactive viewer Dendroscope, version 3.2.10, build 19 (Huson et al., 2007).

To search for protein homologs with known functions, we used the PaperBLAST web tool (Price and Arkin, 2017) and the following parameters: an e-value ≤e^-20^, a protein identity ≥30%, and a query coverage ≥40%. Additionally, functional annotations of the analyzed genes were performed using the UniProt (Magrane and Consortium, 2011), KEGG (Kanehisa et al., 2012), and MetaCyc (Caspi et al., 2014) databases. To clarify the taxonomic affiliations of the analyzed genomes, the NCBI Taxonomy database (http://www.ncbi.nlm.nih.gov/taxonomy) was used.

## Results

The aim of this study was to investigate the nature and distribution of BMCs across 646 microbes commonly found in the HGM (**Supplementary Table S1**). No genes for carboxysomes or ethanol-utilizing BMCs could be found. On the other hand, the analyzed genomes contained all four previously known metabolosomes for the utilization of propanediol, ethanolamine, choline, and fucose or rhamnose (**Figure 2**). Additionally, we reconstructed two novel BMC-associated pathways, utilization of 1-amino-2-propanol/1-amino-2-propanone and xanthine (see the section Novel BMC-Associated Pathways in the HGM). Genes for these two BMCs have been described previously (Axen et al., 2014), but the corresponding metabolic pathways remained unknown. Here, we predicted the pathways for these BMCs and analyzed their distribution in the HGM genomes. Taken together, 103 various functional roles were found to be associated with the BMCs in the analyzed genomes (**Supplementary Table S3**). This systematic analysis provides an unprecedented insight into the BMC distribution in the human gut.

**Figure 3:**
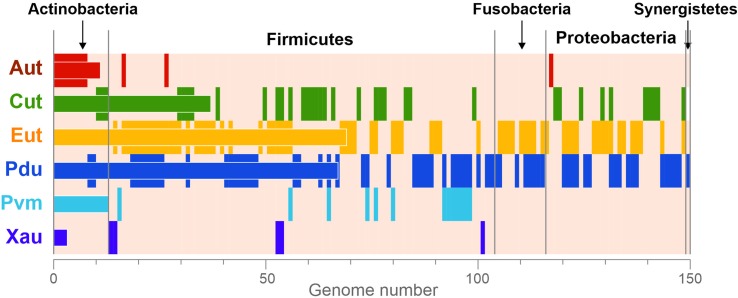
Phyletic distribution of the BMCs. The data are shown only for genomes with at least one BMC. The vertical stripes correspond to the presence of BMCs in a certain genome. The horizontal bars correspond to the total number of BMCs in the analyzed genomes. Aut, 1-amino-2-propanol/1-amino-2-propanone utilization; Cut, choline utilization; Eut, ethanolamine utilization; Pdu, 1,2-propanediol utilization; Pvm, fucose/rhamnose utilization; Xau, xanthine utilization.

Overall, the BMCs were found in 150 (23.2%) analyzed genomes (**Figure 3**), which is in agreement with previous estimations (Cheng et al., 2008; Beeby et al., 2009; Tanaka et al., 2009). All the BMCs were found in the genomes of Actinobacteria (13 genomes, 13.1%), Firmicutes (91 genomes, 30.4%), Fusobacteria (12 genomes, 70.6%), Proteobacteria (33 genomes, 26.8%), and Synergistetes (one genome). No BMCs were found in archaea and also not in the bacterial phyla Bacteroidetes, Spirochaetes, Tenericutes, and Verrucomicrobia (**Supplementary Table S1**). These results suggest that the BMCs are limited to certain phyla within the human gut.

### Previously Known Metabolosomes in the HGM

All four previously known metabolosomes were found in the analyzed genomes. Only one of them, fucose or rhamnose utilization, was phylum specific. This metabolosome was found in 13 (2%) genomes, all belonging to Firmicutes (**Supplementary Table S4**). On the other hand, metabolosomes for the utilization of ethanolamine, 1,2-propanediol, and choline were found in multiple phyla, as described below.

#### Ethanolamine Utilization

The ethanolamine-utilizing (Eut) BMC is the most distributed BMC among the analyzed genomes (**Supplementary Table S5**). It was found in 70 (10.7%) genomes belonging to the phyla Firmicutes, Fusobacteria, and Proteobacteria (**Supplementary Figure S1**). In Firmicutes, the Eut BMC is broadly distributed and is present in genomes from nine families; however, in Proteobacteria, the Eut BMC is a specific feature of the Enterobacteriaceae family (**Supplementary Table S1**). Additionally, ethanolamine ammonia-lyase (EC 4.3.1.7), not associated with the BMC, was found in 39 of 646 (6%) of the analyzed genomes, all belonging to the phyla Actinobacteria, Firmicutes, and Proteobacteria. Thus, Actinobacteria have no Eut utilization BMC but have the signature enzyme.

Previously, two forms of the ethanolamine transporters, EutH (Faust et al., 1990; Stojiljkovic et al., 1995; Kofoid et al., 1999) and Eat (Tsoy et al., 2009), have been described. Both these transporters were found in the analyzed genomes. EutH was found in 81 (12.5%) genomes, all belonging to Actinobacteria, Firmicutes, and Proteobacteria, whereas Eat was found in 12 (1.9%) genomes, belonging to Actinobacteria and Proteobacteria. Additionally, we predicted the additional ethanolamine transporter, EatA, belonging to the ChrA family (Pfam02417). In our HMG genomes, this transporter was only found only in *Ralstonia* sp. 5_7_47FAA and was also co-localized with genes for an ethanolamine ammonia-lyase. The same co-localization could be found in *Ralstonia pickettii* 12D and *R. pickettii* 12J, which were not within our set of HMG genomes.

Three forms of a phosphate acetyltransferase (EC 2.3.1.8) associated with the Eut BMC were found in the analyzed genomes. The first form, previously described as EutD protein in *S. enterica* (Huseby and Roth, 2013; Aussignargues et al., 2015), belongs to the phosphate acetyl/butyryl transferase family (Pfam01515). Among the analyzed genomes, this enzyme form was only found in the genomes of the Enterobacteriaceae family. The second form is similar to PduL protein for the 1,2-propanediol-utilizing BMC and belongs to the phosphate propanoyltransferase family (Pfam06130). This enzyme form was found in 41 genomes of Firmicutes as well as in the genome of *Fusobacterium varium* ATCC 27725. This finding is consistent with the previous results (Tsoy et al., 2009). The third form of the enzyme was predicted in this study by its presence in the Eut BMC gene cluster in 12 genomes of *Fusobacteria* spp., lacking the other genes for a phosphate acetyltransferase. This form belongs to the HAD family (Pfam12710) and demonstrates 28% sequence identity with the phosphoethanolamine/phosphocholine phosphatase (EC 3.1.3.75) from *Pseudomonas aeruginosa* PAO1 (Domenech et al., 2011).

#### Utilization



The 1,2-propanediol-utilizing (Pdu) BMC was found in 67 (10.4%) of the analyzed genomes, all belonging to the phyla Actinobacteria, Firmicutes, Fusobacteria, and Proteobacteria as well as in one of two analyzed Synergistetes genomes. A form of the propanediol dehydratase, which was not associated with the Pdu BMC, was found in six (0.9%) genomes, all belonging to the phyla Actinobacteria and Firmicutes (**Supplementary Table S6**).

The Pdu BMC can use two different types of the signature enzyme propanediol dehydratase: 1) a vitamin B12-dependent enzyme (Bobik et al., 1997; Sriramulu et al., 2008; Parsons et al., 2010) and 2) a B12-independent form (Zarzycki et al., 2017; Levin and Balskus, 2018). The BMC-associated B12-dependent enzyme was found in the 56 (8.7%) genomes. An additional form of the B12-dependent enzyme, which was not associated with the Pdu BMC, was found in 6 of these 56 genomes, belonging to *Citrobacter* spp. (three strains), *Klebsiella* spp. (two strains), and *Yersinia kristensenii*. Surprisingly, in the genome of *Clostridium methylpentosum* DSM 5476, the gene for the B12-dependent enzyme is co-localized with genes for the fucose/rhamnose-utilizing BMC (**Supplementary Figure S1**).

**Figure 4:**
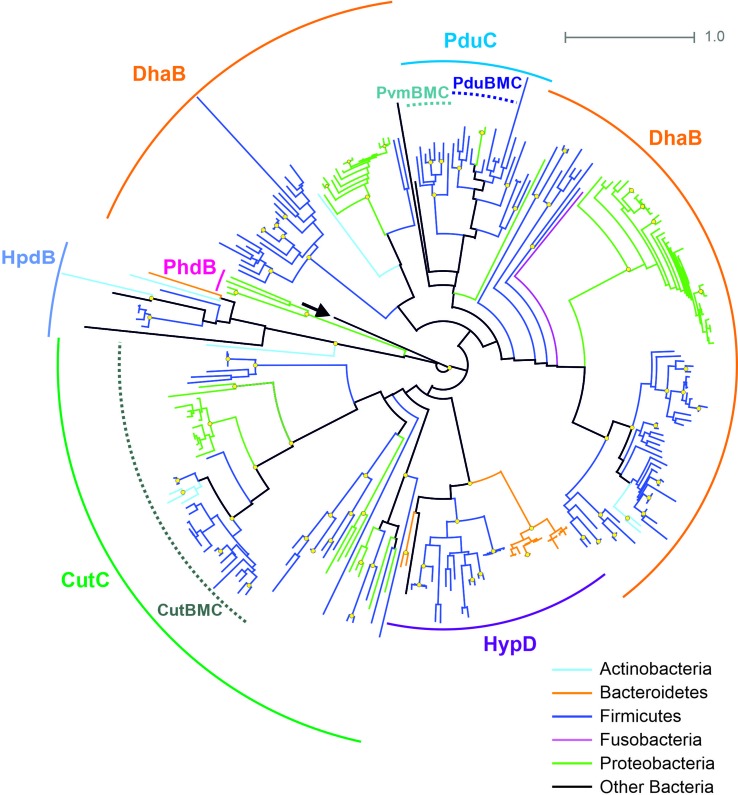
Maximal-likelihood tree for the BMC-associated proteins from the GRE family and their homologs. Branches are painted by microbial phyla. The pyruvate formate-lyase (PflB, EC 2.3.1.54) from *E. coli* is used as outgroup to root the tree and is denoted by an arrow. Bootstrap replicates equal to 100 are marked by yellow circles. Functions of the proteins shown by solid circular arcs: CutC, choline trimethylamine-lyase (EC 4.3.99.4), large subunit; DhaB, glycerol dehydratase (EC 4.2.1.30), B12-independent, large subunit; HpdB, 4-hydroxyphenylacetate decarboxylase (EC 4.1.1.83), large subunit; HypD, 4-hydroxyproline dehydratase (EC 4.2.1.171), large subunit; PduC, (EC 4.2.1.28), B12-independent, large subunit; PhdB, phenylacetate decarboxylase (EC 4.1.1.-), large subunit. Information on previously known proteins with these functions is provided in a **Supplementary Table S2**. BMC-associated enzymes are shown by dotted circular arcs; CutBMC, choline utilization; PduBMC, 1,2-propanediol utilization; PvmBMC, fucose/rhamnose utilization.

The B12-independent form of the propanediol dehydratase belongs to the glycyl radical enzyme (GRE) family, which is highly distributed in microbial genomes, including the HGM ones (Levin et al., 2017; Beller et al., 2018). In addition to B12-independent propanediol dehydratase, the GRE family also includes a choline trimethylamine-lyase, a signature enzyme for the choline-utilizing BMC (Zarzycki et al., 2015; Backman et al., 2017; Zarzycki et al., 2017; Levin and Balskus, 2018). Thus, for the correct prediction of the BMC-associated pathways, a preliminary analysis of the GRE proteins was performed (**Figure 4**). The GRE family propanediol dehydratases were found in the 20 (3.1%) genomes, and, in 14 of the 20, this enzyme was associated with the Pdu BMC. In the further six genomes, this enzyme was associated with the fucose/rhamnose-utilizing BMC.

#### Choline Utilization

The choline-utilizing (Cut) BMC was found in 37 (5.7%) genomes, belonging to the phyla Actinobacteria, Firmicutes, and Proteobacteria. The form of choline trimethylamine-lyase, not associated with the Cut BMC, was found in 21 (3.3%) genomes belonging to the phyla Firmicutes and Proteobacteria (**Supplementary Table S7**, **Figure 4**).

Two choline transporters, LicB and BetT, have been described (Kapatral et al., 2002; Fan et al., 2003). Among the analyzed genomes, LicB was found in 28 (4.3%) genomes, all belonging to Actinobacteria, Firmicutes, and Proteobacteria, whereas BetT was found in 16 (2.5%) genomes, belonging to Firmicutes and Proteobacteria. Additionally, we predicted a novel TRAP-like choline transporter, which was identified through its colocalization with the genes for the choline trimethylamine-lyase in the genomes of *Bilophila wadsworthia* 3_1_6 and *Desulfovibrio* sp. 3_1_syn3. We found the same co-localization in further 11 genomes of *Desulfovibrio* spp., currently not in the HGM set.

### Novel BMC-Associated Pathways in the HGM

An analysis of possible genes for the BMC shell revealed two conserved gene clusters that did not correspond to any known BMC-associated pathway. These clusters have been previously observed (Axen et al., 2014), and some of their enzymes have been experimentally characterized (Kataoka et al., 2006; Kataoka et al., 2008; Mallette and Kimber, 2018). However, the metabolic pathways remain unclear. In this study, we described a distribution of these genes in the HGM genomes and predicted the corresponding metabolic pathways.

#### Utilization



A possible BMC gene cluster with the gene for 1-amino-2-propanol dehydrogenase has been previously described in *Rhodococcus erythropolis* and *Mycobacterium smegmatis* (Urano et al., 2011; Mallette and Kimber, 2018). The enzymatic activity of 1-amino-2-propanol dehydrogenase has been experimentally confirmed (Kataoka et al., 2006; Kataoka et al., 2008), and the transcription of this gene cluster has been shown to be activated by 1-amino-2-propanol (Urano et al., 2011).

**Figure 5:**
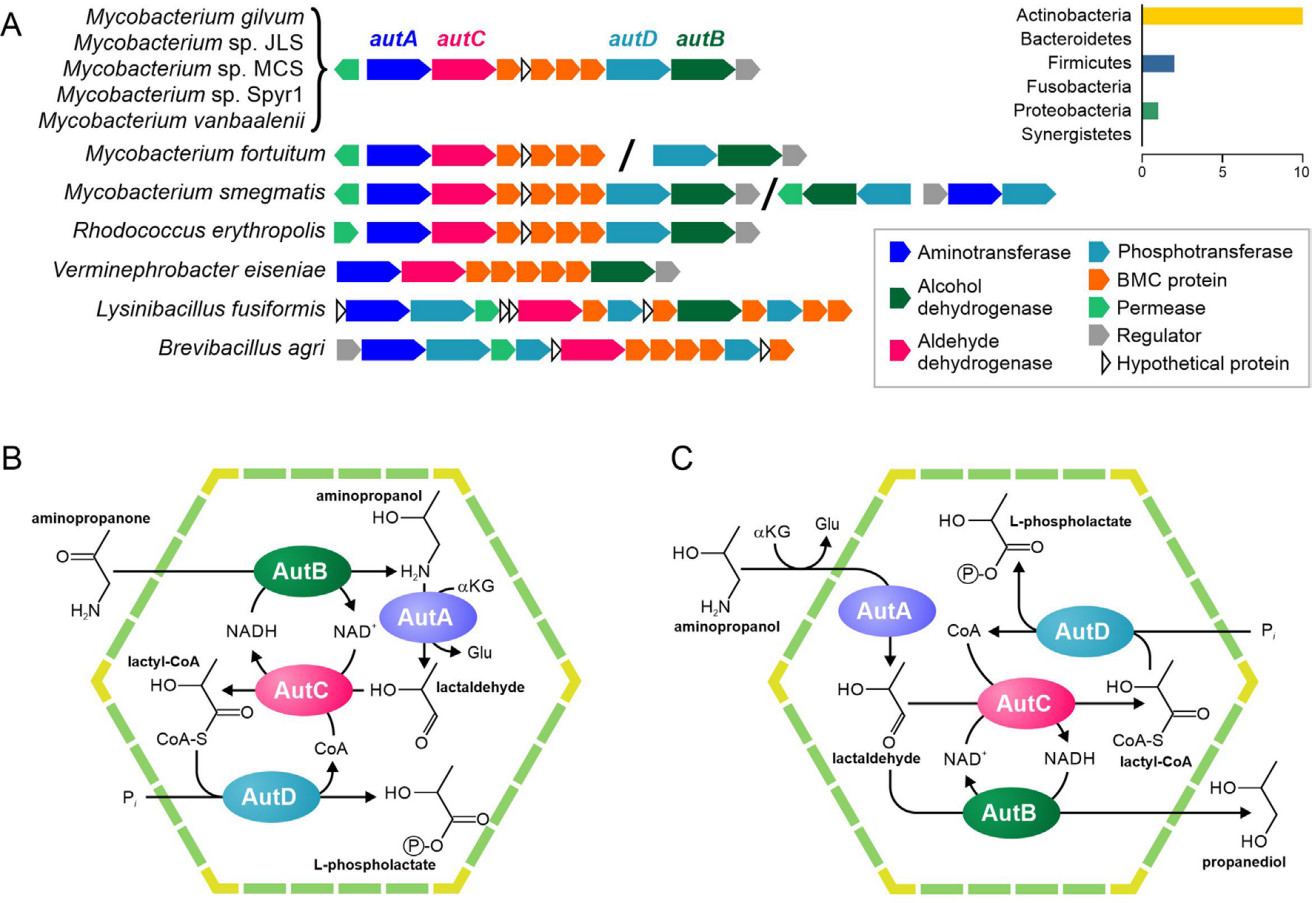
Predicted pathways for the BMC-associated 1-amino-2-propanol/1-amino-2-propanone utilization. **(A)** Locus structure and phyletic distribution of the BMC genes. Distantly located genes are separated by slashes. **(B–C)** Predicted pathways for 1-amino-2-propanol/1-amino-2-propanone utilization.

Among the analyzed genomes, this gene cluster was found in 11 (1.7%) genomes, belonging to the phyla Actinobacteria, Firmicutes, and Proteobacteria (**Supplementary Table S8**, **Figure 5A**). In addition to the BMC shell genes and the gene for 1-amino-2-propanol dehydrogenase, this cluster also contained genes for a GntR family transcriptional regulator (RHOER0001_5064 in *R. erythropolis* SK121), a GabP family permease (RHOER0001_5063), an aminotransferase (RHOER0001_5062), an aldehyde/alcohol dehydrogenase (RHOER0001_5061), and a possible phosphotransferase (RHOER0001_5055). To reconstruct the BMC pathways, we analyzed sequences for all the enzymes encoded in this cluster.

Our analysis of RHOER0001_5062 revealed that this protein had similarity with various aminotransferases (**Supplementary Table S9**), which agreed with the previous prediction (Axen et al., 2014), but had no experimentally characterized orthologs. Thus, we propose that the protein is an aminotransferase of 1-amino-2-propanol or of its derivative, 1-amino-2-propanone. The protein RHOER0001_5055 has no homologs, for which the function is known. Hence, we analyzed its domain structure demonstrated and found similarity to the APH phosphotransferase family (Pfam01636). Surprisingly, the aldehyde/alcohol dehydrogenase (RHOER0001_5061) demonstrated a significant similarity to known BMC enzymes, especially to acetaldehyde dehydrogenases from the Eut and Cut BMCs (**Supplementary Figure S2**). Thus, we propose that RHOER0001_5061 protein encodes an enzyme that converts an aldehyde to an acyl-CoA. Based on the available experimental data and predicted functions of the enzymes, we proposed that this gene cluster encodes a 1-amino-2-propanol/1-amino-2-propanone utilization (Aut) BMC similar to the metabolosome.

Based on available experimental data and the predicted functions of the genes for the Aut BMC, we propose two possible scenarios for an associated pathway. In the first scenario (**Figure 5A**), the 1-amino-2-propanone is reduced to 1-amino-2-propanol by the 1-amino-2-propanol dehydrogenase (AutB). Next, the aminotransferase (AutA) would convert 1-amino-2-propanol to lactaldehyde. In turn, lactaldehyde would be transformed to lactyl-CoA by the aldehyde dehydrogenase (AutC) and then to L-phospholactate by the phosphotransferase (AutD). In this scenario, NADH would be produced by the AutC and would be utilized by the AutB, so the NAD^+^/NADH ratio inside the BMC would be maintained. This scenario is based on the proposition that the *aut* gene cluster can be transcriptionally activated not only by 1-amino-2-propanol but also by 1-amino-2-propanone, which is quite speculated.

In a second scenario (**Figure 5D**), AutA would also convert 1-amino-2-propanol to lactaldehyde, which would be further converted to lactyl-CoA by the AutC and then to L-phospholactate by the AutD. However, unlike the previous scenario, AutB reduces lactaldehyde to 1,2-propanediol. As in the first scenario, NADH would be produced by the AutC and utilized by the AutB. Nonetheless, this scenario would require AutB to be bifunctional enzyme with 1-amino-2-propanol dehydrogenase and lactaldehyde dehydrogenase activities, which is not very likely.

#### Xanthine Utilization

**Figure 6:**
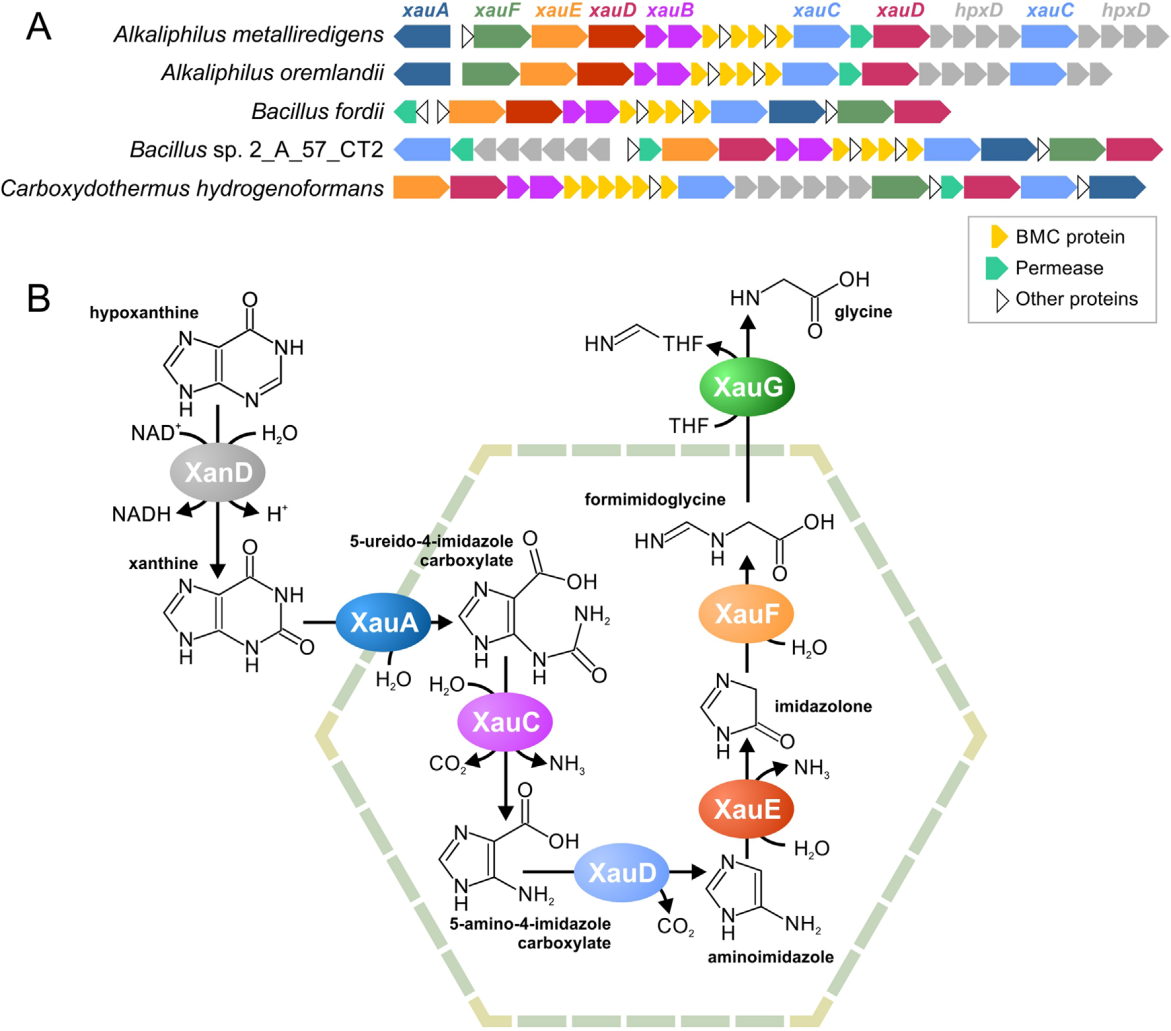
Predicted BMC for xanthine utilization, a locus structure **(A)** and a proposed pathway **(B)**. THF, tetrahydrofolate.

The previously described gene cluster for the BMC of unknown function (Axen et al., 2014) was found in five analyzed genomes belonging to the Firmicutes (**Figure 6A**, **Supplementary Table S10**). Together with proteins of a BMC shell, a part of this cluster was conserved in multiple genomes and includes a permease and various enzymes. These proteins were checked for the existence of homologs with known function (**Table S9**). Based on reactions, catalyzed by those homologs (**Supplementary Figure S3**), we predicted the functions of the proteins in this cluster as follows. 1) The *Amet_4569-68* gene cluster encodes a dehydrogenase of aromatic nitrogen-containing compounds. 2) The *Amet_4587* and *Amet_4584* genes encode hydrolases that can disrupt aromatic rings. The *Amet_4587*-encoded enzyme most probably can hydrolase only rings of six atoms, whereas the *Amet_4584*-encoded enzyme can be specific for six or five atoms ring. 3) The *Amet_4583* and *Amet_4572* genes encode amidohydrolases. 4) The *Amet_4581* gene encodes a decarboxylase of aromatic compounds. 5) Finally, the *Amet_4586* gene encodes a formimidoyltransferase.

Based on these predicted functions, we proposed that this pathway degrades nicotinamide, purines, or pyrimidines. Because pyrimidines and nicotinamide have only one aromatic ring, while there are two different hydrolases in the gene cluster, we proposed that this pathway is likeliest to degrade purine nucleotides. Two pathways are known for such a process, an anaerobic pathway (Pope et al., 2009) and an anaerobic pathway (Pricer and Rabinowitz, 1956; Vogels and Van Der Drift, 1976). Because the aerobic pathway requires the oxidative opening of the aromatic ring and no such reactions was encoded by homologs of the analyzed proteins, we propose the pathway to be for anaerobic xanthine utilization (Xau, **Figure 6B**). Unfortunately, no genes for the anaerobic degradation of xanthine have been previously described; thus, we cannot compare our predictions with any experimental data. The proteins encoded by the *Amet_4569-68* genes demonstrate a significant similarity to known xanthine dedydrogenases (**Table S9**). Thus, we propose that Amet_4569-68 (XanD) is a xanthine dehydrogenase (XanD), representing the first step of the pathway. Among the two ring-opening hydrolases, only the Amet_4584 has homologs that hydrolyze a five-atom ring. Thus, this protein was considered as the enzyme for the sixth step of the pathway (XauF), whereas another hydrolase, Amet_4587 (XauA), was considered as the enzyme for the second step. For the third step of the pathway (XauC), the amidohydrolase separating carbamoyl group is required. The only protein with homologs harboring such activity is Amet_4587 (XauA). Thus, another aminohydrolase, Amet_4572 (XauE), was considered to be responsible for the fifth step of the pathway. The protein Amet_4581 (XauD) was the only decarboxylase among the analyzed proteins; thus, it was considered to be the fourth step of the pathway. Similarly, the only transferase, Amet_4586 (XauG), was considered as the seventh and final step of the pathway. Thus, in the predicted pathway, each molecule of xanthine is degraded into two molecules of ammonia, two molecules of carbon dioxide, and one molecule of formimidoglycine.

Genes for XanD are present in all genomes with the *xau* gene cluster but are co-localized only in four of the five genomes (**Figure 6A**). Thus, we propose that the XanD-encoded step would take place outside the Xau BMC. Additionally, the last step of the pathway requires a transfer of tetrahydrofolate. Because tetrahydrofolate is a large molecule, the last reaction should also be outside the BMC. This proposal is supported by the absence of a gene for this enzyme in the Xau BMC gene cluster in the genomes of *Bacillus fordii* DSM 16014 and *Bacillus* sp. 2_A_57_CT2.

## Discussion

In this study, we conducted a systematic analysis of the distribution of BMCs across 646 genomes of human gut microbes using comparative genomic approaches. For the previously known BMCs, three non-orthologous displacements were found. These displacements include a novel form of the enzyme, phosphate acetyltransferase for the ethanolamine utilization in Fusobacteria, as well as novel transporters for an ethanolamine and a choline. Additionally, two novel BMC-associated pathways were predicted demonstrated the value of comparative genomics.

### Novel BMC-Associated Pathways

We predicted two novel metabolic pathways for the previously defined loci (Axen et al., 2014). For the utilization of 1-amino-2-propanol/1-amino-2-propanone (Aut), we suggest two possible scenarios (see the section 1-Amino-2-Propanol/1-Amino-2-Propanone Utilization). However, to establish the correct mechanism, further experiments will have to be performed. For instance, an activation of the *aut* operon by 1-amino-2-propanone would support the first scenario, whereas a confirmation of second enzymatic activity of lactaldehyde dehydrogenase for AutB would support the second one. Thus, this study provides testable hypotheses to establish the biochemical function of the Aut BMC.

The second reconstructed pathway is for the utilization of xanthine (Xau), in which no one enzyme has been experimentally characterized. Using comparative genomics, we were able to identify this pathway as anaerobic xanthine degradation. We also reconstructed the sequence of reactions based on reaction steps found in some Firmicutes (Pricer and Rabinowitz, 1956) but no genomic sequences are available for these strains. Moreover, no gene for the anaerobic xanthine degradation is known. Thus, this predicted pathway also requires experimental validation.

Previously, two functional paradigms for BMCs have been formulated, anabolic carboxysomes and catabolic metabolosomes. The Aut BMC corresponds to the metabolosome paradigm because the both proposed scenarios (**Figure 5D, E**) consist of the same types of reactions as all other metabolosomes (**Figure 2**), differing only in the order of these reactions. In contrast, the Xau pathway contains a completely different composition of enzymes. Thus, we hypothesize that there is a third functional paradigm for BMCs.

We predicted one additional BMC gene cluster in the genome *Clostridium hylemonae* DSM 15053. In contrast to the *aut* and *xau* gene clusters, this cluster has never been reported. It contains genes for BMC shell proteins (CLOHYLEM_04359-60 and CLOHYLEM_04362), transporters (CLOHYLEM_04347 and CLOHYLEM_04350), hydrolases (CLOHYLEM_04349, CLOHYLEM_04357, and CLOHYLEM_04361), dehydrogenase (CLOHYLEM_04353-56), NDP-forming acyl-CoA ligase (CLOHYLEM_04351-52), and O-acyltransferase (CLOHYLEM_04345). Such a set of functions differs from that for carboxysomes, metabolosomes, or the Xau BMC, which indicates that there may be one more functional paradigm. Unfortunately, we could find this cluster only in the genome *C. hylemonae*, making it impossible to analyze it using a comparative genomic approach. We hope that as new genome sequences become available, the function of this gene cluster will be further clarified.

### A Role of the 1,2-Propanediol Dehydratase in the Fucose/Rhamnose Utilization

Since the discovery of the Pvm BMC, two pathway scenarios have been proposed. A first scenario (Erbilgin et al., 2014) proposes that one molecule of lactaldehyde, formed by the breakage of fuculose or rhamnulose phosphate, is reduced to 1,2-propanediol, whereas another molecule is oxidized to lactyl-CoA, which, in turn, is transformed to lactate (**Figure 1E**). A second scenario (Petit et al., 2013; Zarzycki et al., 2015) proposes that lactaldehyde is reduced to 1,2-propanediol, which is further transformed to propionaldehyde by the propanediol dehydratase. Propanediol is further transformed to propionate by the same pathway as that in the Pdu BMC. To select between these scenarios, we analyzed the genomic context of the genes for Pvm BMC and propanediol dehydratase (PduCD). The Pvm BMC was present in 13 analyzed genomes, while the PduCD was present only in 10 of these 13 genomes and co-localized with the BMC genes only in six of 13 genomes. Thus, we conclude that the Pvm pathway corresponds to the first scenario, which did not include the PduCD reaction. The chromosomal co-localization of the PduCD genes with the Pvm BMC gene cluster in some genomes can be explained by 1,2-propanediol being a product of the Pvm pathway and by a functional coupling of these two pathways.

Taken together, comparative genomics techniques allowed us to analyze of the BMC distribution and to uncover novel BMC loci. Additionally, comparative genomics can in resolving questions related to BMC-associated metabolic pathways.

### Evolution of the BMCs

The BMCs for different metabolic pathways can have multiple similar proteins, not only shell proteins but also enzymes. Thus, annotation of the BMC gene clusters requires accuracy as well as the use of multiple analysis methods. On the other hand, the presence of similar enzymes in different BMCs allowed us to identify certain patterns in the evolution of the analyzed BMCs.

#### Evolution of the Ethanolamine Utilization

A broad distribution of Eut BMC and the ethanolamine ammonia-lyase, not associated with BMC, has been demonstrated (Tsoy et al., 2009) Additionally, an evolutionary scenario has been proposed, in which Eut BMC appeared in Firmicutes, and then the corresponding gene cluster was horizontally transferred to the ancestral genomes of Fusobacteria and Enterobacteriaceae family of Proteobacteria (Tsoy et al., 2009). The present analysis of the ethanolamine utilization in the HGM genomes confirmed a possible horizontal transfer of the Eut BMC gene cluster from Firmicutes to Fusobacteria. Accordingly, on the phylogenetic tree for the heavy chain of ethanolamine ammonia-lyase, the branch corresponding to the Fusobacteria was located inside the branch for the BMC-associated proteins from Firmicutes (**Supplementary Figure S1**). The same positions of the Fusobacteria branches on the phylogenetic trees were observed for aldehyde dehydrogenase and acetate/propionate kinases (**Supplementary Figure S2**). Most of the analyzed Fusobacteria contained phosphate acetyltransferases from the HAD family, whereas the *F. varium* contained two PduL-like phosphate acetyltransferases (see the section Evolution of the Ethanolamine Utilization). At the phylogenetic tree for BMC-associated acyltransferases (**Supplementary Figure S2C**), these PduL-like proteins were also located inside the branch corresponding to the Firmicutes phosphate acetyltransferases, which was associated with the Eut BMC. It appears that the Eut BMC gene cluster, which was transferred from Firmicutes to Fusobacteria, lacked a gene for alcohol dehydrogenase. Consequently, the branch for Eut BMC-associated alcohol dehydrogenases from Fusobacteria was not located inside the branch for this enzyme in Firmicutes, but clusters together with the branch for Pdu BMC-associated alcohol dehydrogenases from the same phylum (**Supplementary Figure S2**).

The results of this study call into question the previous hypothesis of horizontal gene transfer of the Eut BMC genes from Firmicutes to Enterobacteriaceae. We propose an alternative hypothesis in which the Eut BMC either would be a common ancestor of Firmicutes and Enterobacteriaceae or would have appeared twice, independently in each of these phyla. This hypothesis is supported by as follows. 1) At the phylogenetic trees for the heavy chain of ethanolamine ammonia-lyase, aldehyde dehydrogenases, acetate kinases, and alcohol dehydrogenases, branches for the Enterobacteriaceae proteins were separated from the branches for the Firmicutes proteins (**Supplementary Figures S1–S2**). 2) The PduL-like form of the phosphate acetyltransferase (see 3.3.1) was not found in any of the analyzed Enterobacteriaceae genomes.

Another interesting observation regarding the evolution of the ethanolamine utilization concerns the acetate kinases in the Firmicutes, which, in some cases, were located together with the propionate kinases in a branch (**Supplementary Figure S2**). Most probably, this branch corresponds to the bifunctional enzymes, acetate/propionate kinases, and genes for these enzymes may be co-localized on the chromosome with Eut or Pdu BMCs. Because acetate and propionate kinases are not encapsulated into BMCs (Kerfeld et al., 2010; Zarzycki et al., 2015; Kerfeld et al., 2018), there are no additional limits, such as encapsulation into the proper BMC, because this enzyme participates in the utilization of both 1,2-propanediol and ethanolamine.

#### Evolution of the 1,2-Propanediol Utilization

The Pdu BMC pathway seems to appear in Firmicutes and was then horizontally transferred to microorganisms of other taxa. At the phylogenetic trees for all the Pdu enzymes (**Supplementary Figures S1–S2**), the branches of proteins from Proteobacteria were located inside the branches of proteins from Firmicutes. Moreover, it seems that such a gene transfer occurred at least twice 1) to the common ancestor of Enterobacteriaceae and 2) to the common ancestor of *Escherichia* sp. 3_2_53FAA and *Escherichia hermannii* NBRC 105704. Most of the Enterobacteriaceae had a B12-dependent form of propanediol dehydratase, whereas these two strains of *Escherichia* spp. had a GRE family form of this enzyme (**Supplementary Table S6**). Additionally, Pdu BMC proteins of these two *Escherichia* spp. were clustered with the proteins from Clostridiales, such as *Anaerococcus* spp., *Eubacterium* spp., *Faecalibacterium* spp., *Flavonifractor* spp., and *Ruminococcus* spp. On the other hand, the Pdu proteins for the most part of Enterobacteriaceae were clustered on the trees with the proteins from Lactobacillales, such as *Enterococcus* spp., *Lactobacillus* spp., and *Listeria* spp. It appears that the Pdu gene cluster from Clostridiales was also transferred to other groups of HGM organisms, such as to Actinobacteria (*Propionibacterium freudenreichii* CIRM-BIA1 and *Propionibacterium propionicum* F0230a), Fusobacteria, and Synergistetes (*Anaerobaculum hydrogeniformans* ATCC BAA-1850). Consistently, branches for the proteins from these organisms were located inside or clustered with the branches for the proteins from Clostridiales (**Supplementary Figures S1–S2**).

#### Evolution of Choline Utilization

The Cut BMC could have appeared from a common ancestor of Firmicutes and Proteobacteria or independently in each of these phyla. Accordingly, in all the phylogenetic trees (**Figure 3**, **Supplementary Figure S2**), the Cut proteins from Proteobacteria clustered apart from the proteins from Firmicutes. On the other hand, the Cut proteins from some Actinobacteria (*Atopobium minutum* 10063974, *Collinsella tanakaei* YIT 12063, and *Olsenella uli* DSM 7084) were located inside the branches for the proteins from Firmicutes (**Figure 3**, **Supplementary Figures S1–S2**). Thus, we can see that the same group of species actively acted as a gene donor in a horizontal gene transfer, donating Cut genes to Actinobacteria as well as Pdu genes to *A. hydrogeniformans*, *Escherichia* spp., *Fusobacteria* spp., and *Propionibacterium* spp. Such an active gene transfer may be explained by 1) an abundance of these Firmicutes species in the HGM and by 2) a benefit from an acceptance of BMC genes.

#### Evolution of the 1-amino-2-propanol/1-amino-2-Propanone Utilization

The genes for the Aut BMC were found in a small number of genomes. However, unlike other rare BMCs, e.g., Pvm and Xau, the Aut BMC was not taxon-specific. In fact, the Aut BMC was found in the genomes of three phyla, Actinobacteria, Firmicutes, and Proteobacteria, whereas Pvm and Xau BMC were found only in Firmicutes. Thus, an evolution of the Aut BMC may be of exceptional interest in relation to a BMC origin and evolution.

The permease proteins (AutP) form two distantly related branches at the phylogenetic tree; one branch corresponds to proteins from Firmicutes, whereas another includes proteins from Actinobacteria and Proteobacteria (**Supplementary Figure S5**). The BMC-associated aminotransferases (AutA) and dehydrogenases (AutB) demonstrate a similar phylogeny. On the phylogenetic trees, both AutA and AutB formed a monophyletic branch containing all the BMC-associated proteins from the phyla Actinobacteria, Firmicutes, and Proteobacteria (**Supplementary Figure S5**). The CoA-transferring aldehyde dehydrogenase proteins (AutC) were similar to the acetaldehyde dehydrogenases from the Eut and Cut BMCs (**Supplementary Figure S2**). Similar to the AutP proteins, the AutC from Actinobacteria and Proteobacteria clustered together, whereas the proteins from Firmicutes formed a separate branch distantly related to them. The gene for the phosphotransferase (AutD) was not present in the *aut* gene cluster in Proteobacteria. However, in Firmicutes, this gene cluster contained at least two copies of the *autD* genes. At the phylogenetic tree, the AutD proteins from Firmicutes form two branches; one of them clustered together with the AutD from Actinobacteria, whereas the other was distantly related to the latter (**Supplementary Figure S5**). In Actinobacteria, the Aut BMC was found in eight genomes. However, in Firmicutes and Proteobacteria, it was found only in one and two genomes, respectively. We conclude that Aut BMC appeared in the Actinobacteria and was then transferred to Proteobacteria, *Verminephrobacter eiseniae* EF01-2, or its ancestor. Additionally, a part of this gene cluster, without the permease, was transferred to Firmicutes, namely to the common ancestor of *Brevibacillus agri* BAB-2500 and *Lysinibacillus fusiformis* ZB2. The permease protein, as well an additional copy of AutC, could appear in Firmicutes independently, by convergent evolution.

Generally, the following trends in the evolution of all analyzed BMCs may be noted. 1) BMCs appeared most likely in the common ancestor of Proteobacteria and Firmicutes but were then lost in multiple taxa. 2) BMC gene clusters are often subjects of horizontal gene transfer. Thus, both Eut and Pdu BMCs were transferred from Firmicutes to Fusobacteria. 3) A horizontal gene transfer sometimes does not involve all the genes for a certain BMC, which results in non-orthologous displacements, similar to the appearance of a new phosphate acetyltransferase in Fusobacteria. 4) In all the trees for the common components of the metabolosomes (**Supplementary Figure S2**), Cut and Eut proteins form branches, close to each other. Likely, one of these BMCs is an ancestor to another, especially since these BMCs differ only in their signature enzymes.

### Concluding Remarks

Starting from the initial objective to systematically analyze the distribution of BMC in the HGM genomes, this study resulted in unexpected findings, such as the reconstruction of two previously unknown pathways, one of which being for anaerobic xanthine degradation. We consequently propose a third functional paradigm for the BMCs, in addition to the anabolic carboxysome and catabolic metabolosome.

The results of this study connect gut microbes to host health, nutrition, and disease. The analyzed BMCs can utilize ethanolamine and 1,2-propanediol, which are associated with food poisoning (Korbel et al., 2005), or produce trimethylamine, which is associated with kidney and cardiovascular diseases (Moraes et al., 2015; Tang et al., 2015; Rath et al., 2017; Ashikhmin et al., 2018). Additionally, BMCs can participate in the utilization of fucose and rhamnose, regular dietary-derived carbohydrates (Petit et al., 2013; Erbilgin et al., 2014; Zarzycki et al., 2015). As a next step, one could compare metagenomic data for the healthy and diseased subject to analyze differences in the level of genes for the BMC-associated pathways, which may serve as health/disease markers in medical diagnostics.

Additionally, metabolites, which are degraded or produced by BMCs, may differ between individuals, which may be predicted using computational modeling of microbiome metabolic models (Thiele et al., 2013; Magnusdottir and Thiele, 2017) and an individual’s metagenomic data (Baldini et al., 2018). For instance, computational modeling of bile acids biotransformation demonstrated that HGM communities of healthy individuals and patients with inflammatory bowel disease differ in their capability to synthesize certain metabolites but that this capability is not a direct read-out of gene abundance (Heinken et al., 2019). Thus, the modeling of the BMC-associated metabolism for various HGM communities may help us to identify non-trivial microbial metabolites, which may impact a person’s health state.

An analysis of BMCs beyond the HGM genomes may lead to the discovery of novel pathways, similar to known or even completely different from the existing functional paradigms. Such novel BMCs may be of particular interest for molecular engineering (Axen et al., 2014; Plegaria and Kerfeld, 2017; Kerfeld et al., 2018), and as such substantially advance a synthetic biology and other areas of biotechnology.

## Data Availability Statement

The datasets analyzed for this study can be found in the PubSEED database (http://pubseed.theseed.org; the subsystem name is “Bacterial Microcompartments (BMC) HGM”). The protein sequences for the annotated genes in the FASTA format are represented in the file Sequences S1 in the Supplementary Materials.

## Author Contributions

DR and IT conceived of and designed the research project. DR, IT, and LM wrote the manuscript. DR, LM, and SS performed the genomic analysis of the BMC pathways. All authors read and approved the final manuscript.

## Funding

This study was funded by the Luxembourg National Research Fund (FNR) through the CORE program grant (C16/BM/11332722 to DR) and by the European Research Council (ERC) under the European Union’s Horizon 2020 research and innovation program (grant agreement No 757922).

## Conflict of Interest Statement

The authors declare that the research was conducted in the absence of any commercial or financial relationships that could be construed as a potential conflict of interest.

## Supplementary Material

Figure S1 Maximal-likelihood trees for the signature enzymes in ethanolamine and 1,2-propanediol utilization; **(A)** ethanolamine ammonia-lyase heavy chain (EutB), **(B)** large subunit of the B12-dependent propanediol dehydratase (PduC^B12^). The trees are rooted at midpoints, and the roots are shown with arrows. Branches are painted by microbial phyla. Dotted circular arcs show the BMC-associated enzymes. Bootstrap replicates (n = 100) are marked by yellow circles.

Figure S2 Maximal-likelihood trees for the metabolosome core enzymes: **(A)** CoA-acylating aldehyde dehydrogenases (AutC, CutB, CutF, EutE, PduP, and PvmJ); **(B)** acetate/propionate kinases (CutQ, CutS, EutP, EutQ, PduV, PduW, and PvmG); **(C)** phosphate acyltransferases (pfam06130 only; CutH, EutD, PduL, and PvmB); **(D)** alcohol dehydrogenases (CutO, EutE, EutG, PduQ, and PvmO). The trees are rooted at midpoints, and the roots are shown by arrows. Branches are painted by microbial phyla. Specificities of the BMCs are shown by solid circular arcs: Aut, 1-amino-2-propanol/1-amino-2-propanone utilization; Cut, choline utilization; Eut, ethanolamine utilization; Pdu, 1,2-propanediol utilization; Pvm, fucose/rhamnose utilization. Bootstrap replicates equal to 100 are marked by yellow circles.

Figure S3 Predicted pathway for xanthine utilization and reactions catalyzed by homologous experimentally analyzed proteins (for details see Supplementary Table S9). Locus tags are shown for the genome of *Alkaliphilus metalliredigens* QYMF.

Figure S4 Alternative pathway predicted for Pvm BMC based on (Zarzycki et al., 2015).

Figure S5 Maximal-likelihood trees for the proteins involved in the 1-amino-2-propanol/1-amino-2-propanone utilization pathway: **(A)** permeases (AutP) and their homologs; **(B)** aminotransferases (AutA) and their homologs; **(C)** dehydrogenases (AutB) and their homologs; **(D)** phosphotransferases (AutD) and their homologs. The trees are rooted at midpoints; arrows show the roots. The branches are painted by microbial phyla. Dotted circular arcs show BMC-associated proteins. Bootstrap replicates equal to 100 are marked by yellow circles.

Table S1 List of the analyzed genomes. ^(1)^ Genome status, finished (F) or draft (D). ^(2)^ Reason for the inclusion of the genome. ^(3)^ Presence of BMC and non-BMC signature enzyme: “-”, BMC is absent; “BMC”, BMC is present; “nBMC”, only the non-compartmentalized copy of the signature enzyme is present; “BMC + nBMC”, both of the last forms are present.

Table S2 Previously known proteins for the analyzed BMCs.

Table S3 Functions of the proteins and genes analyzed in this work.

Table S4 Presence of genes for fucose/rhamnose utilization in the analyzed genomes. The PubSEED identifiers are shown. For details on the analyzed organisms, see Supplementary Table S1. For details on gene functions, see Supplementary Table S3. Information for propanediol utilization (for details see Table S5) is added to demonstrate their co-presence with the genes for fucose/rhamnose utilization.

Table S5 Presence of genes for ethanolamine utilization in the analyzed genomes. The PubSEED identifiers are shown. For details on the analyzed organisms, see Supplementary Table S1. For details on gene functions, see Supplementary Table S3.

Table S6 Presence of genes for 1,2-propanediol utilization in the analyzed genomes. The PubSEED identifiers are shown. For details on analyzed organisms, see Supplementary Table S1. For details on gene functions, see Supplementary Table S3.

Table S7 Presence of genes for choline utilization in the analyzed genomes. The PubSEED identifiers are shown. For details on analyzed organisms, see Supplementary Table S1. For details on gene functions, see Supplementary Table S3.

Table S8 Presence of genes for 1-amino-2-propanol/1-amino-2-propanone utilization in the analyzed genomes. The PubSEED identifiers are shown. For details on analyzed organisms, see Supplementary Table S1. For details on gene functions, see Supplementary Table S3.

Table S9 The results for the search of functionally analyzed homologs for novel proteins using the PaperBLAST tool.

Table S10 Presence of genes for xanthine utilization in the analyzed genomes. The PubSEED identifiers are shown. For details on the analyzed organisms, see Supplementary Table S1. For details on gene functions, see Supplementary Table S3.

Sequences S1 FASTA format protein sequences for all the proteins annotated in this work.
