# Supplementary material for: Comparative Genomic Analysis Reveals Novel Microcompartment-Associated Metabolic Pathways in the Human Gut Microbiome
Source: Front Genet. 2019 Jul 4;10:636. doi: 10.3389/fgene.2019.00636 (PMC6620236; doi:10.3389/fgene.2019.00636)

Figure S1. Maximal-likelihood trees for the signature enzymes in ethanolamine and 1,2-propanediol utilization; (A) ethanolamine ammonia-lyase heavy chain (EutB), (B) large subunit of the B12-dependent propanediol dehydratase ($PduC^{B12}$). The trees are rooted at midpoints, and the roots are shown by arrows. Branches are painted by microbial phyla. Dotted circular arcs show the BMC-associated enzymes. Bootstrap replicates equal to 100 are marked by yellow circles.

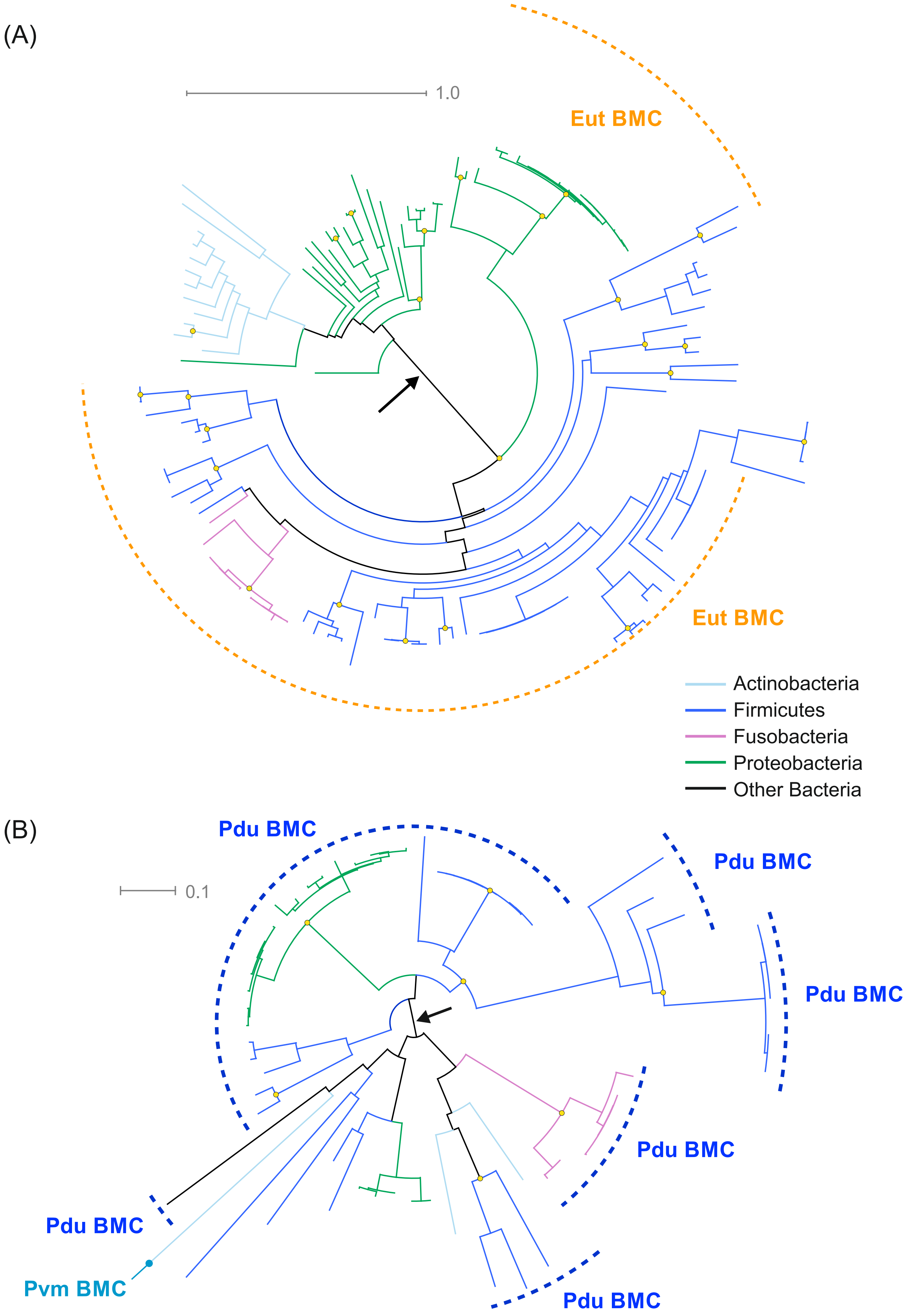

Supplement: Figure S1 — Maximal-likelihood trees for the signature enzymes in ethanolamine and 1,2-propanediol utilization; (A) ethanolamine ammonia-lyase heavy chain (EutB), (B) large subunit of the B12-dependent propanediol dehydratase (PduCB12). The trees are rooted at midpoints, and the roots are shown with arrows. Branches are painted by microbial phyla. Dotted circular arcs show the BMC-associated enzymes. Bootstrap replicates (n = 100) are marked by yellow circles. [file DataSheet_1.pdf]
