# Supplementary material for: Comparative Genomic Analysis Reveals Novel Microcompartment-Associated Metabolic Pathways in the Human Gut Microbiome
Source: Front Genet. 2019 Jul 4;10:636. doi: 10.3389/fgene.2019.00636 (PMC6620236; doi:10.3389/fgene.2019.00636)

Figure S2. Maximal-likelihood trees for the metabolosome core enzymes: (A) CoA-acylating aldehyde dehydrogenases (AutC, CutB, CutF, EutE, PduP, and PvmJ); (B) acetate/propionate kinases (CutQ, CutS, EutP, EutQ, PduV, PduW, and PvmG); (C) phosphate acyltransferases (pfam06130 only; CutH, EutD, PduL, and PvmB); (D) alcohol dehydrogenases (CutO, EutE, EutG, PduQ, and PvmO). The trees are rooted at midpoints, and the roots are shown by arrows. Branches are painted by microbial phyla. Specificities of the BMCs are shown by solid circular arcs: Apu, 1-amino-2-propanol/1-amino-2-propanone utilization; Cut, choline utilization; Eut, ethanolamine utilization; Pdu, 1,2-propanediol utilization; Pvm, fucose/rhamnose utilization. Bootstrap replicates equal to 100 are marked by yellow circles.

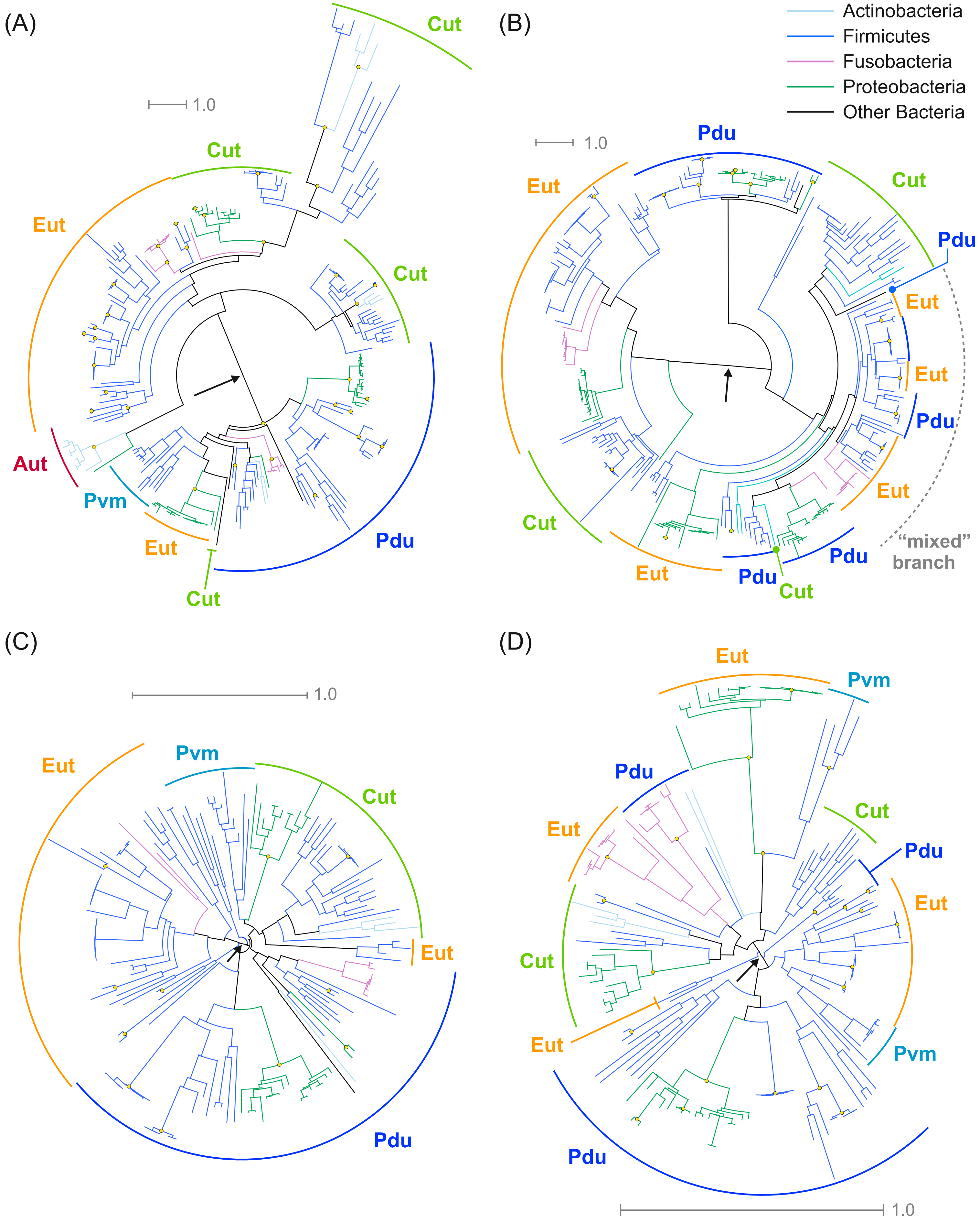

Supplement: Figure S2 — Maximal-likelihood trees for the metabolosome core enzymes: (A) CoA-acylating aldehyde dehydrogenases (AutC, CutB, CutF, EutE, PduP, and PvmJ); (B) acetate/propionate kinases (CutQ, CutS, EutP, EutQ, PduV, PduW, and PvmG); (C) phosphate acyltransferases (pfam06130 only; CutH, EutD, PduL, and PvmB); (D) alcohol dehydrogenases (CutO, EutE, EutG, PduQ, and PvmO). The trees are rooted at midpoints, and the roots are shown by arrows. Branches are painted by microbial phyla. Specificities of the BMCs are shown by solid circular arcs: Aut, 1-amino-2-propanol/1-amino-2-propanone utilization; Cut, choline utilization; Eut, ethanolamine utilization; Pdu, 1,2-propanediol utilization; Pvm, fucose/rhamnose utilization. Bootstrap replicates equal to 100 are marked by yellow circles. [file DataSheet_2.pdf]
