# Supplementary material for: Comparative Genomic Analysis Reveals Novel Microcompartment-Associated Metabolic Pathways in the Human Gut Microbiome
Source: Front Genet. 2019 Jul 4;10:636. doi: 10.3389/fgene.2019.00636 (PMC6620236; doi:10.3389/fgene.2019.00636)

Figure S3. Predicted pathway for xanthine utilization and reactions catalyzed by homologous experimentally analyzed proteins (for details see Supplementary Table S9). Locus tags are shown for the genome of *Alkaliphilus metalliredigens* QYMF.

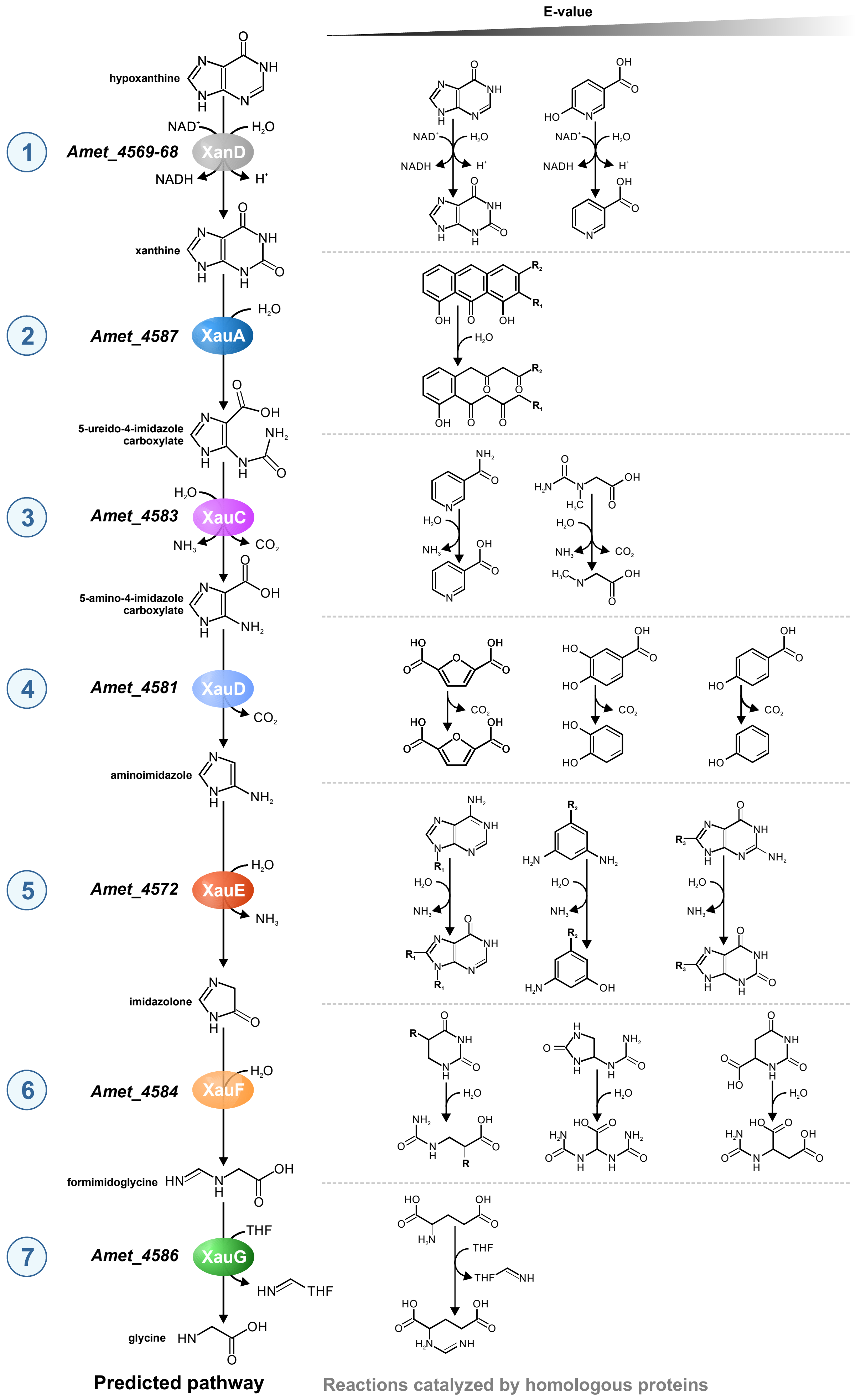

Supplement: Figure S3 — Predicted pathway for xanthine utilization and reactions catalyzed by homologous experimentally analyzed proteins (for details see Supplementary Table S9). Locus tags are shown for the genome of Alkaliphilus metalliredigens QYMF. [file DataSheet_3.pdf]
