# Supplementary figures and images for: Comparative Genomic Analysis Reveals Novel Microcompartment-Associated Metabolic Pathways in the Human Gut Microbiome
Source: Front Genet. 2019 Jul 4;10:636. doi: 10.3389/fgene.2019.00636 (PMC6620236; doi:10.3389/fgene.2019.00636)

Figure S4. Alternative pathway predicted for Pvm BMC, by (Zarzycki et al., 2015).

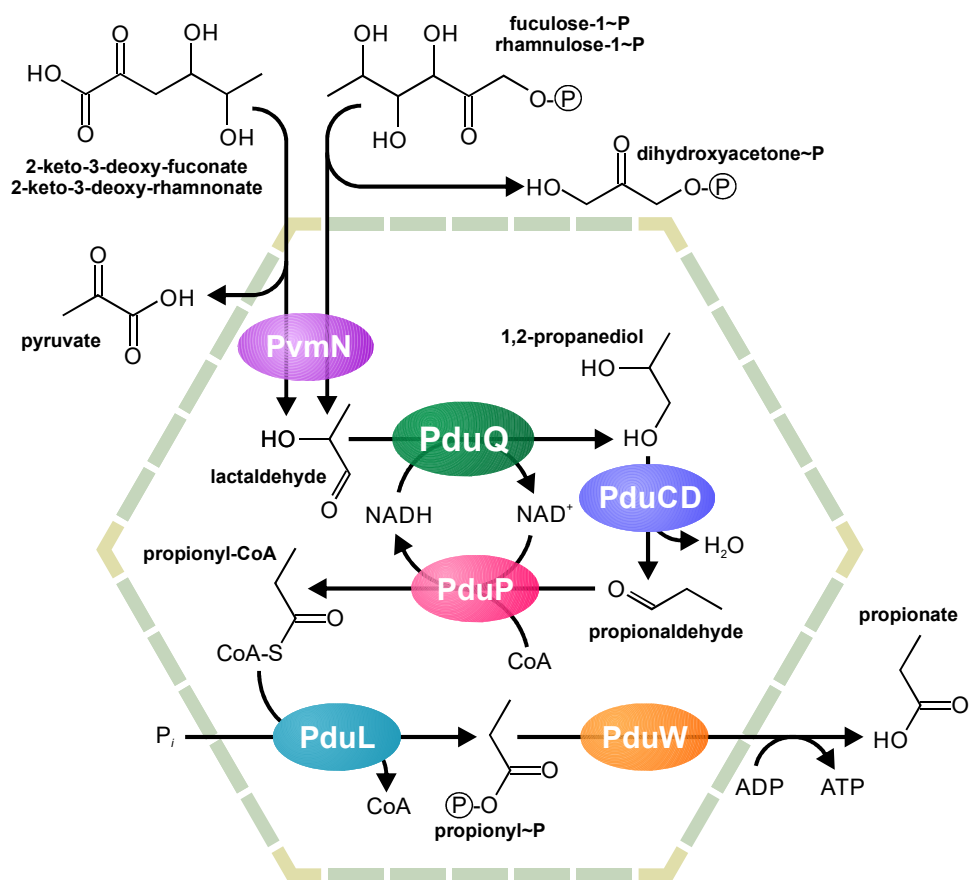

Supplement: Figure S4 — Alternative pathway predicted for Pvm BMC based on (Zarzycki et al., 2015). [file DataSheet_4.pdf]
