# Supplementary material for: Comparative Genomic Analysis Reveals Novel Microcompartment-Associated Metabolic Pathways in the Human Gut Microbiome
Source: Front Genet. 2019 Jul 4;10:636. doi: 10.3389/fgene.2019.00636 (PMC6620236; doi:10.3389/fgene.2019.00636)

Figure S5. Maximal-likelihood trees for the proteins involved in the 1-amino-2-propanol/1-amino-2-propanone utilization pathway: (A) permeases (AutP) and their homologs; (B) aminotransferases (AutA) and their homologs; (C) dehydrogenases (AutB) and their homologs; (D) phosphotransferases (AutD) and their homologs. The trees are rooted at midpoints; arrows show the roots. The branches are painted by microbial phyla. Dotted circular arcs show BMC-associated proteins. Bootstrap replicates equal to 100 are marked by yellow circles.

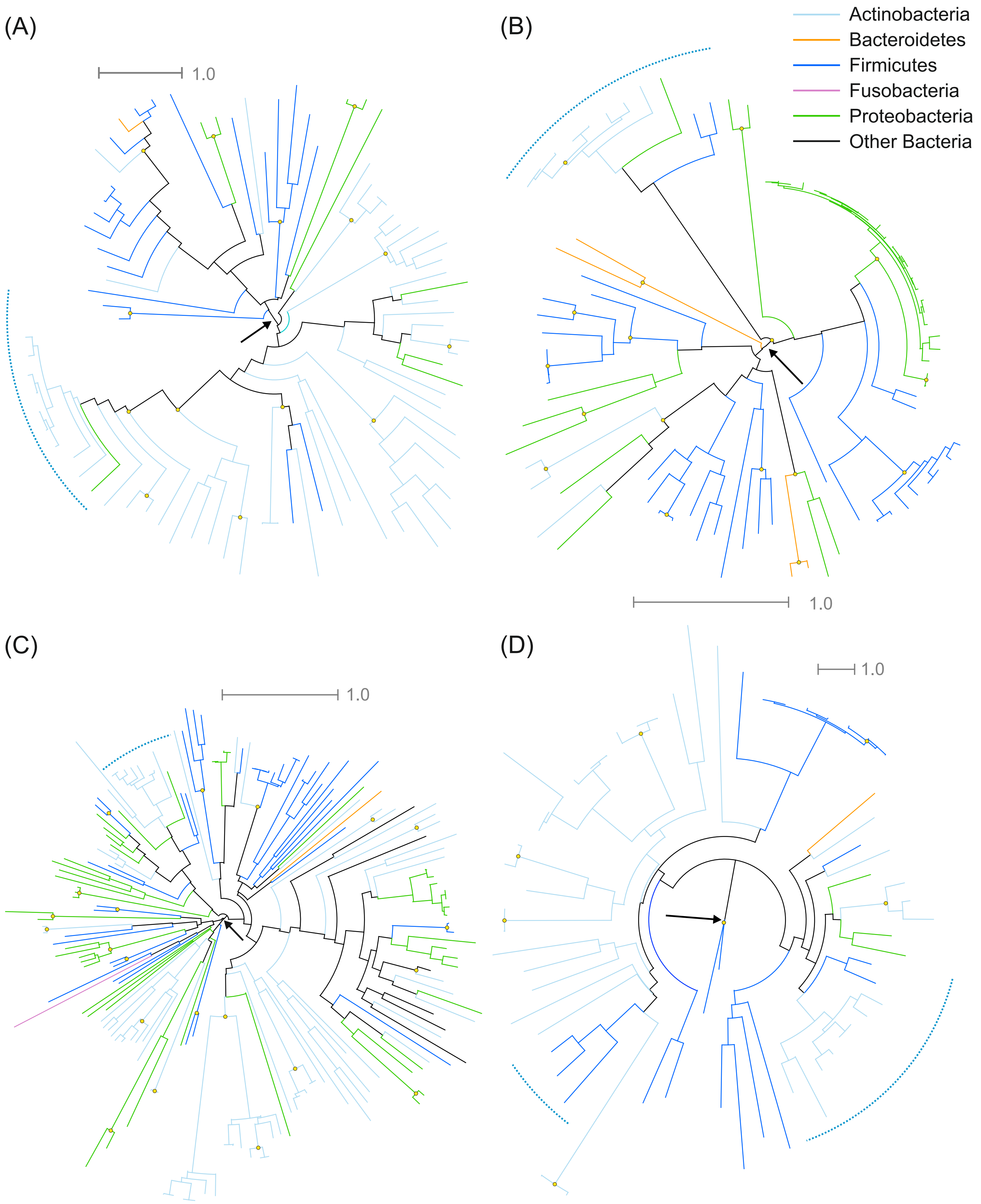

Supplement: Figure S5 — Maximal-likelihood trees for the proteins involved in the 1-amino-2-propanol/1-amino-2-propanone utilization pathway: (A) permeases (AutP) and their homologs; (B) aminotransferases (AutA) and their homologs; (C) dehydrogenases (AutB) and their homologs; (D) phosphotransferases (AutD) and their homologs. The trees are rooted at midpoints; arrows show the roots. The branches are painted by microbial phyla. Dotted circular arcs show BMC-associated proteins. Bootstrap replicates equal to 100 are marked by yellow circles. [file DataSheet_5.pdf]
